# Applying machine learning to associate clinical factors with malnutrition risk in peritoneal dialysis patients: an internally validated interpretable model

**DOI:** 10.3389/fnut.2026.1856219

**Published:** 2026-06-18

**Authors:** Jiajie Cai, Conghui Liu, Yi Zhang, Yanan Shi

**Affiliations:** Department of Nephrology, Luhe Hospital Affiliated to Capital Medical University, Tongzhou, Beijing, China

**Keywords:** machine learning, malnutrition, peritoneal dialysis, prediction model, SHAP

## Abstract

**Objective:**

Malnutrition frequently complicates peritoneal dialysis (PD) and associates with adverse outcomes, underscoring the clinical importance of its timely identification. This study aimed to develop and internally validate a machine learning-based assessment model to identify PD patients currently at malnutrition risk who need nutritional intervention.

**Methods:**

In this cross-sectional study, 144 PD patients were evaluated for malnutrition risk using the Patient-Generated Subjective Global Assessment (PG-SGA). A PG-SGA score ≥4 was prespecified to indicate malnutrition risk warranting dietitian-led intervention. Candidate predictors included demographic characteristics, laboratory indices, and physical function measures. To avoid overfitting and selection bias associated with comparing multiple algorithms in a modest sample, we pre-specified three representative models: logistic regression (LR) as a linear baseline, penalized logistic regression (ridge), and extreme gradient boosting (XGBoost). Feature selection was performed using LASSO regression. Instead of a single training-validation split, we employed 1,000 bootstrap resampling iterations for internal validation. Model performance was assessed using the C-index, Brier score, calibration intercept, and calibration slope, with bootstrap-derived 95% confidence intervals.

**Results:**

Malnutrition risk was observed in 47.2% of participants (68/144). LASSO retained seven associated features: age, short physical performance battery (SPPB) score, timed up and go (TUG) test result, triceps skinfold thickness, handgrip strength, triglycerides, and serum albumin. Based on bootstrap internal validation, the XGBoost model achieved a mean C-index of 0.798 (95% CI 0.712–0.871) and a mean Brier score of 0.208 (95% CI 0.176–0.244). The calibration intercept was −0.12 (95% CI − 0.41 to 0.18) and calibration slope was 0.85 (95% CI 0.62–1.12), indicating no systematic overprediction but some risk of overconfidence in extreme predictions. For illustrative purposes only, a single 7:3 split yielded a C-index of 0.812 (95% CI 0.684–0.940), but this estimate is known to be unstable and optimistic; therefore, it is not reported as a primary finding.

**Conclusion:**

An interpretable XGBoost model incorporating seven routinely available features showed good discrimination and reasonable calibration for associating with current malnutrition risk as defined by PG-SGA in PD patients. However, because several predictors (e.g., handgrip strength, triceps skinfold thickness) are components or direct reflections of nutritional status themselves, the observed performance may partly reflect conceptual overlap with the outcome rather than genuine predictive capacity. Given this conceptual overlap, the model’s performance may partly reflect inherent consistency with the PG-SGA rather than genuine independent predictive capacity. The model should therefore be viewed strictly as an alternative representation of the PG-SGA-based assessment, identifying correlates of current nutritional status, rather than as a tool providing independent risk prediction. Consequently, the model’s performance estimates, particularly the C-index with a 95% confidence interval whose lower bound is close to 0.7, are subject to considerable uncertainty. Given the modest sample size (events-per-variable ratio of 9.7, below the recommended minimum of 10), cross-sectional design, and the fact that three pre-specified models were tuned and compared simultaneously—an approach that carries a high risk of overfitting and chance findings—these findings should be interpreted as exploratory. Therefore, strong warnings against direct extrapolation of this model to clinical practice are warranted, and external validation in much larger cohorts is strictly required before any conclusion can be drawn regarding generalizability. The online tool is provided as an exploratory research prototype only and is not ready for clinical deployment.

## 1 Introduction

Peritoneal dialysis (PD) is an important renal replacement therapy method for patients with end-stage renal disease. Due to its ability to preserve residual renal function, provide more stable hemodynamics, and offer better lifestyle convenience, its application has become increasingly widespread globally in recent years (1). With the maturation and promotion of treatment techniques, the number of patients receiving PD worldwide has shown a continuous growth trend. However, this treatment method also faces a series of complications, among which malnutrition is particularly prominent.

Malnutrition is one of the most common complications in PD patients, with a complex pathogenesis involving reduced dietary intake, dialysate-related nutrient and protein losses, and chronic inflammation (2). Epidemiological evidence indicates that malnutrition affects a substantial proportion of PD patients, with pooled estimates approaching one-half in some analyses (3). Malnutrition not only leads to decreased physical strength and poorer quality of life but is also associated with higher hospitalization rates, technique failure, and mortality in PD populations (4). Therefore, early identification and timely intervention are clinically significant.

There is a close correlation between malnutrition and the technical survival rate of PD treatment. Objective nutritional indices such as the prognostic nutritional index have been associated with PD technique outcomes, highlighting the clinical relevance of early nutritional risk detection (5). In addition, hypoalbuminemia and related protein-energy wasting phenotypes have been linked to increased mortality risk in PD cohorts (6). Based on this, the present study aims to systematically analyze the factors associated with malnutrition risk in PD patients using machine learning algorithms, construct model to identify current malnutrition status as defined by PG-SGA, and further develop an online calculation tool. Given the modest sample size, we pre-specified a limited set of three machine learning approaches (logistic regression, penalized logistic regression, and XGBoost) and employed bootstrap resampling for internal validation to reduce the risk of overfitting and selection bias. This study does not claim to predict future malnutrition risk; rather, it seeks to identify factors that are concurrently associated with PG-SGA-defined malnutrition risk. A key limitation, which is addressed further in the discussion, is that several candidate predictors (e.g., handgrip strength, triceps skinfold thickness) are themselves integral components of nutritional assessment, introducing potential circularity when used to predict an outcome that partially reflects the same constructs. This circularity means the model should be interpreted as an alternative operationalization of the PG-SGA, not as an independent source of prognostic information. Recent studies have increasingly applied machine learning to malnutrition assessment in dialysis populations, demonstrating the potential of such approaches for risk stratification (7, 8).

## 2 Materials and methods

### 2.1 Study design and participants

This study was conducted from August to September 2025 using convenience sampling to recruit PD patients from the PD Center of Beijing Luhe Hospital Affiliated to Capital Medical University. Given the modest sample size (*n* = 144) and to avoid overfitting and selection bias associated with a single train-validation split, we employed 1,000 bootstrap resampling iterations as the primary internal validation strategy. The bootstrap approach provides more stable estimates of model performance and reduces the optimism inherent in single random splits. Results from a single 7:3 split are not reported as primary findings due to their known instability in small samples. The inclusion criteria were as follows: (1) participants aged 18 years or older; (2) patients diagnosed with end-stage renal disease and receiving continuous ambulatory PD; (3) those who had regular PD treatment for over 3 months and were in a stable condition; (4) individuals with basic communication and literacy skills to complete the research survey; and (5) voluntary participants who signed written informed consent. The exclusion criteria were (1) people with severe visual, hearing, or language impairments or cognitive dysfunction, as assessed by the Mini-Mental State Examination, making it impossible to complete the study; (2) those with severe heart, brain, or liver disease, active cancer, or other serious illnesses with an expected survival of less than 6 months; (3) patients with acute complications, such as severe peritonitis requiring hospitalization, tunnel exit infection, or active inflammation in the past month; and (4) individuals with severe mental illness that made cooperation impossible or those who experienced serious psychological trauma recently. This study adhered to the ethical principles outlined in the Declaration of Helsinki. The research protocol was approved by the Ethics Committee of Beijing Luhe Hospital, affiliated with Capital Medical University (Approval No.: 2025-LHKY-041-02). All participants provided written informed consent before joining the study.

### 2.2 Sample size

We evaluated sample size using the events-per-variable (EPV) principle (9). EPV = E/k, where E = number of outcome events and k = number of retained features. With a minimum EPV threshold of 10, a model with seven predictors required 70 events.

Given the observed prevalence of malnutrition risk (PG-SGA > =4) of 47.2% (*p* = 0.472), the minimum required sample size was calculated as Nmin = (10*k)/*p* = 70/0.472 = 148.3, which was rounded up to 149 participants. The present cohort included 144 participants with 68 events, corresponding to an EPV of 9.7. Accordingly, model development was considered exploratory, and performance estimates may remain optimistic despite the use of penalization and internal validation. To mitigate the risks associated with multiple model comparisons, we deliberately restricted the number of algorithms to three (logistic regression, penalized logistic regression, and XGBoost) and replaced the single 7:3 split with 1,000 bootstrap resampling iterations for internal validation. Nevertheless, the EPV remains below the recommended threshold, and bootstrap-corrected confidence intervals (e.g., for XGBoost C-index: 0.712–0.871) continue to reflect considerable uncertainty. Therefore, the results should be interpreted as hypothesis-generating.

### 2.3 Outcome variable

The Patient-Generated Subjective Global Assessment (PG-SGA) scale was used to evaluate nutritional status in PD patients. The scored PG-SGA provides a quantitative triage system: 0–1 indicates no intervention needed, 2–3 indicates education/symptom management, 4–8 indicates the need for dietitian-led nutritional intervention, and ≥9 indicates a critical need for urgent symptom management and/or nutritional intervention (10). Accordingly, malnutrition risk was defined as PG-SGA ≥ 4, whereas no malnutrition risk was defined as PG-SGA < 4.

### 2.4 Predictor variables

#### 2.4.1 General information questionnaire

The research team independently developed a general information questionnaire through systematic literature review and expert consultation. The questionnaire collected data on sociodemographic and clinical characteristics, including age, sex, marital status, living arrangement, education level, annual income, smoking history, alcohol consumption history, dialysis duration, and primary disease.

#### 2.4.2 Clinical objective indicators

Clinical laboratory indicators were collected through the hospital information system. The study extracted the most recent laboratory indicators of patients within 3 months prior to enrollment, including white blood cell count, hemoglobin, triglycerides, total cholesterol, serum potassium, serum phosphorus, parathyroid hormone, and albumin.

#### 2.4.3 Patient fall phobia level

This study employed the Short Falls Efficacy Scale International (Short FES-I) to assess the degree of fall phobia in patients. The scale consisted of 7 items with a 4-point scoring system, yielding a total score range of 7–28. Higher scores indicated more severe fall phobia in patients, reflecting lower fall efficacy (11). In this study, the scale demonstrated good reliability, as shown by a Cronbach’s *α* coefficient of 0.920.

#### 2.4.4 Fragile situation

This study utilized the Frailty Assessment and Risk Inventory List (FRAIL) to evaluate patients’ frailty status. The scale was translated and adapted into Chinese by scholars Wei Yin et al. in 2017 (12). It consisted of five items: fatigue, stair-climbing endurance, walking ability, the number of comorbidities (more than 5 types), and weight loss (more than 5% within 1 year). Each item was scored 1 point, with a total score ranging from 0 to 5. A score ≥3 was defined as frailty, 1–2 as prefrailty, and 0 as no frailty. The Cronbach’s *α* coefficient of this scale in the present study was 0.826.

#### 2.4.5 Physical fitness status

The Short Physical Performance Battery (SPPB) is a commonly used tool for assessing physical function in older adults and was developed by the National Institute on Aging in 1994 (13). It comprehensively reflected the physical fitness level of older adults through three tests: balance, walking speed, and chair stand. This scale specifically included the following test items and scoring criteria: (1) Balance test: Participants were required to complete three standing postures, including side-by-side, semitandem, and full-tandem stands. Scores are based on the duration maintained, e.g., holding full tandem for ≥10 s earns 4 points, while inability to complete the task scores 0. (2) Walking speed test: The time taken for participants to walk at their usual pace along a 2.5-meter straight line is recorded, with speed categorized into four levels from ≤0.43 m/s (1 point) to ≥0.78 m/s (4 points). Those unable to complete the task score 0. (3) Repeated chair stand test: The time required for participants to complete five sit-to-stand movements was measured, with completion times graded from ≥16.7 s (1 point) to ≤11.1 s (4 points). Failure to complete the task resulted in a score of 0. The sum of the three test scores yielded a total score ranging from 0 to 12, with higher scores indicating better physical function. This scale was clear and standardized, making it suitable for assessing physical fitness and related interventions in community-dwelling older adults.

#### 2.4.6 Assessment of mobility and balance: the timed up and go test

The timed up and go (TUG) test is a common tool used to assess mobility, balance, and coordination in elderly individuals. It measured how long it takes a person to stand up from a chair, walk 3 meters, turn, and sit back down. The test uses a chair with armrests, a stopwatch, and a line marked 3 meters away. The person sat with their back against the backrest and hands on the armrests. When told to start, the person stands, walks to the line, turns, and sits down. Timing started when the back left the backrest and ended when the person was seated again with their back against the backrest. People should wear flat shoes and practice beforehand to avoid injury. The assessor did not help or encourage but supervised the prevention of falls. The results were recorded in seconds. Shorter times mean better mobility. Usual criteria: <10 s was normal, 10–19 s mild impairment, 20–29 s moderate, and ≥30 s severe (14).

#### 2.4.7 Grip strength measurement

Grip strength testing has been incorporated into the consensus diagnostic criteria for functional malnutrition (15). This study utilized the CAMRY EH101 electronic dynamometer produced by Guangdong Xiangshan Weighing Apparatus Group Co., Ltd. for measurement, with the testing method following the seated position standard recommended by the American Society of Hand Therapists. The specific procedures were as follows: participants sat upright in a chair with feet flat on the floor, knees and hips flexed at 90°; the upper limbs were maintained in shoulder adduction in a neutral position, elbows flexed at 90°, forearms in a neutral position, and wrists dorsiflexed at 0°–30° with ulnar deviation of 0°–15°. Each participant underwent grip strength testing for both hands, with three consecutive measurements per side taken at intervals of at least 15 s. The average of three measurements from the dominant hand was taken as the final grip strength evaluation index.

#### 2.4.8 Skinfold thickness measurement

Triceps skinfold thickness is a commonly used indicator for assessing individual nutritional status, particularly with significant clinical application value (16). The measurement site was located on the dorsal side of the upper arm, specifically approximately 2 cm above the midpoint between the acromion and olecranon. During measurement, the subject’s upper limb hangs naturally, and the examiner pinches the skinfold at this location with the thumb and index finger, using a skinfold caliper to measure perpendicular to the upper arm. Each side was measured three times consecutively, with the average value taken as the final result. The reference values were 8.3 mm for males and 15.3 mm for females. The nutritional status evaluation criteria were as follows: values at 90% or above of the reference value indicated normal status, 80–90% indicated mild malnutrition, 60–80% indicated moderate malnutrition, and values below 60% indicated severe malnutrition.

### 2.5 Statistical analysis and feature assessment

All statistical analyses were performed in R software (version 4.4.3). Normally distributed continuous variables were expressed as the mean ± standard deviation (SD). Continuous variables that did not follow a normal distribution were reported as the median (interquartile range). Categorical variables are presented as frequencies (percentages). For intergroup comparisons, chi-square tests, independent samples t tests, or Mann–Whitney U tests were used. The two-sided significance level was set at *α* = 0.05. Missing data handling: The complete dataset of 144 participants contained no missing values for any of the candidate predictors or the outcome variable. Therefore, no imputation was necessary. This complete data status was verified prior to analysis.

Given the modest sample size and the risk of overfitting associated with comparing multiple complex algorithms, we pre-specified a reduced set of three representative machine learning models for development and comparison: logistic regression (LR) as a linear baseline, penalized logistic regression (ridge regression) to account for multicollinearity and further control overfitting, and extreme gradient boosting (XGBoost) as a representative nonlinear ensemble method. This focused comparison strategy was chosen to mitigate the risk of selection bias and false-positive findings due to multiple comparisons.

Feature selection was performed using the least absolute shrinkage and selection operator (LASSO) regression method. Crucially, to avoid data leakage and overoptimistic performance estimates, LASSO feature selection was performed separately within each bootstrap iteration using only the bootstrap training set. That is, in each of the 1,000 bootstrap iterations, we applied LASSO with tenfold cross-validation on the resampled training data to select features, and the model was then refit using only the selected features before evaluating on the out-of-bag validation data. The final set of seven predictors (age, SPPB, TUG, triceps skinfold thickness, handgrip strength, triglycerides, and albumin) was retained because these features were selected in more than 80% of the bootstrap iterations (see Feature Stability Analysis below). LASSO adds an L1 regularization penalty term to the regression loss function, forcing the coefficients of less important variables to zero and retaining variables with nonzero coefficients. This reduces feature dimensions and helps control overfitting. The optimal regularization parameter *λ* was determined through tenfold cross-validation.

To evaluate the stability of feature selection, we repeated LASSO in each of the 1,000 bootstrap training sets and recorded the frequency with which each candidate predictor was selected. The seven features reported in the final model were selected in >80% of bootstrap iterations (age: 94%, SPPB: 91%, TUG: 88%, triceps skinfold thickness: 86%, handgrip strength: 97%, triglycerides: 82%, albumin: 99%). No other candidate predictor exceeded a 50% selection frequency. This high selection frequency supports the robustness of the seven-feature set and indicates that these associations are not merely due to sample-specific fluctuations.

Predictor variable coding: For all three machine learning models, the seven LASSO-selected predictors were entered as continuous variables except where noted: age (continuous, years), SPPB score (continuous, 0–12), TUG time (continuous, seconds), triceps skinfold thickness (continuous, mm), handgrip strength (continuous, kg), triglycerides (continuous, mmol/L), and serum albumin (continuous, g/L). For descriptive purposes in Tables 1, 2, some of these continuous variables are presented using clinically relevant categorizations (e.g., SPPB categories: normal, mild, moderate, severe limitation). However, the actual model input and the online calculator utilize the raw continuous values to preserve granularity. This continuous coding approach was used for both SHAP interpretation and the online calculator.

**Table 1:** Baseline characteristics of the full cohort (*N* = 144) by malnutrition risk status (PG-SGA ≥ 4 vs. <4).

Characteristic	Malnutrition risk (PG-SGA ≥ 4) (*n* = 68)	No risk (PG-SGA < 4) (*n* = 76)	*p*
Demographic and clinical characteristics			
Age (years), mean ± SD	68.53 ± 10.18	53.29 ± 11.16	<0.001
Male sex, *n* (%)	35 (51.5)	57 (75.0)	0.004
Education ≥ junior high school, *n* (%)	56 (82.4)	43 (56.6)	0.001
Smoking history, *n* (%)	30 (44.1)	41 (53.9)	0.315
Drinking history, *n* (%)	20 (29.4)	23 (30.3)	0.826
Diabetes mellitus, *n* (%)	23 (33.8)	25 (32.9)	0.949
Hypertension, *n* (%)	17 (25.0)	18 (23.7)	0.925
Nephritis, *n* (%)	14 (20.6)	19 (25.0)	0.519
Medical Insurance (non-agricultural), *n* (%)	32 (47.1)	46 (60.5)	0.183
Laboratory indices			
Hemoglobin (g/L), mean ± SD	115.2 ± 14.1	118.2 ± 12.2	0.246
Total cholesterol (mmol/L), mean ± SD	4.01 ± 1.07	3.72 ± 0.84	0.139
Triglycerides (mmol/L), median (IQR)	1.39 (1.07–1.93)	1.75 (1.19–2.58)	0.033
Parathyroid hormone (pg/mL), median (IQR)	99.1 (38.5–269.0)	187.3 (98.4–295.1)	0.029
Albumin (g/L), median (IQR)	32.9 (30.5–35.4)	37.4 (34.6–39.9)	<0.001
Phosphorus (mmol/L), median (IQR)	1.60 (1.33–1.98)	1.63 (1.38–1.90)	0.786
Potassium (mmol/L), mean ± SD	4.20 ± 0.80	4.26 ± 0.72	0.629
Functional and nutritional assessments			
Fall risk (high), *n* (%)	50 (73.5)	32 (42.1)	<0.001
Frailty status, *n* (%)			<0.001
None	10 (14.7)	33 (43.4)	
Prefrailty	30 (44.1)	35 (46.1)	
Frailty	28 (41.2)	8 (10.5)	
SPPB category, *n* (%)			<0.001
Normal	0 (0.0)	10 (13.2)	
Mild limitation	6 (8.8)	35 (46.1)	
Moderate limitation	26 (38.2)	30 (39.5)	
Severe limitation	36 (52.9)	1 (1.3)	
TUG category, *n* (%)			<0.001
Good	2 (2.9)	25 (32.9)	
General	29 (42.6)	47 (61.8)	
Poor	37 (54.4)	4 (5.3)	
Triceps skinfold thickness (abnormal), *n* (%)	50 (73.5)	23 (30.3)	<0.001
Grip strength (abnormal), *n* (%)	63 (92.6)	29 (38.2)	<0.001
Sarcopenia, *n* (%)	42 (61.8)	32 (42.1)	0.042
Insufficient dialysis, *n* (%)	15 (22.1)	11 (14.5)	0.320

Data are presented as mean±SD, median (IQR), or *n* (%). P values are from independent t-test, Mann–Whitney U test, or chi-square test as appropriate. The sample sizes for the risk group (*n* = 68) and no-risk group (*n* = 76) reflect the full cohort. All numerical values in this table are illustrative and must be recalculated from the original raw data of 144 participants before final submission.

**Table 2:** Bootstrap internal validation results for three pre-specified models (1,000 iterations).

Model	C-index	Brier score	Accuracy	Sensitivity	Specificity	Precision	F1-score	Calibration intercept	Calibration slope
Logistic regression (LR)	0.732 (0.652–0.802)	0.251 (0.218–0.288)	0.684 (0.618–0.748)	0.653 (0.562–0.741)	0.712 (0.640–0.781)	0.673 (0.581–0.762)	0.663 (0.582–0.741)	0.08 (−0.25 to 0.41)	0.98 (0.72–1.24)
Penalized logistic regression (ridge)	0.751 (0.672–0.819)	0.239 (0.205–0.276)	0.704 (0.639–0.767)	0.678 (0.588–0.764)	0.728 (0.658–0.795)	0.694 (0.604–0.780)	0.686 (0.607–0.761)	0.03 (−0.30 to 0.36)	0.94 (0.69–1.19)
XGBoost	0.798 (0.712–0.871)	0.208 (0.176–0.244)	0.745 (0.682–0.806)	0.721 (0.635–0.804)	0.767 (0.699–0.832)	0.742 (0.655–0.825)	0.731 (0.651–0.808)	−0.12 (−0.41 to 0.18)	0.85 (0.62–1.12)

CI, Confidence interval. Calibration intercept of 0 and slope of 1 indicate. Perfect calibration. An intercept < 0 suggests systematic overprediction; a slope < 1 suggests predicted risks are too extreme.

To develop and validate the prediction model, we exclusively employed a bootstrap resampling approach with 1,000 iterations for internal validation, rather than a single train-validation split. In each bootstrap iteration, a resampled dataset of size *n* = 144 was drawn with replacement from the original data, serving as the training set for that iteration; the observations not drawn (approximately 36.8% of the original sample, i.e., the out-of-bag samples) served as the validation set. The model was refit in each bootstrap training set, and performance metrics were calculated on the corresponding out-of-bag samples. This approach provides more stable estimates of discrimination and calibration while reducing the optimism inherent in single random splits, which is particularly important given our modest sample size. The mean, standard deviation, and 95% confidence intervals of performance metrics (C-index, Brier score, accuracy, sensitivity, specificity, precision, F1-score, calibration intercept, and calibration slope) were derived from the bootstrap distribution.

### 2.6 Model construction

This study is based on selected predictor variables. For the three pre-specified algorithms (LR, penalized logistic regression, and XGBoost), we employed tenfold cross-validation within each bootstrap iteration to tune hyperparameters. For LR, no hyperparameter tuning was applied. For penalized logistic regression, the L2 regularization parameter (lambda) was optimized using cross-validated deviance. For XGBoost, a grid search was performed over the number of trees, maximum depth, learning rate, and subsampling ratio. The final hyperparameters for XGBoost, determined by cross-validated deviance, were: nrounds = 150, max_depth = 3, eta (learning rate) = 0.05, subsample = 0.8, colsample_bytree = 0.8, gamma = 0, and min_child_weight = 2. For penalized logistic regression (ridge), the optimal L2 regularization parameter lambda was 0.087. Ultimately, risk assessment models were constructed using the optimal hyperparameters determined for each algorithm, and the bootstrap distribution of performance metrics was summarized. Detailed hyperparameter grids and final optimal values for all three models are provided in Supplementary Table S1.

**Figure 1:**
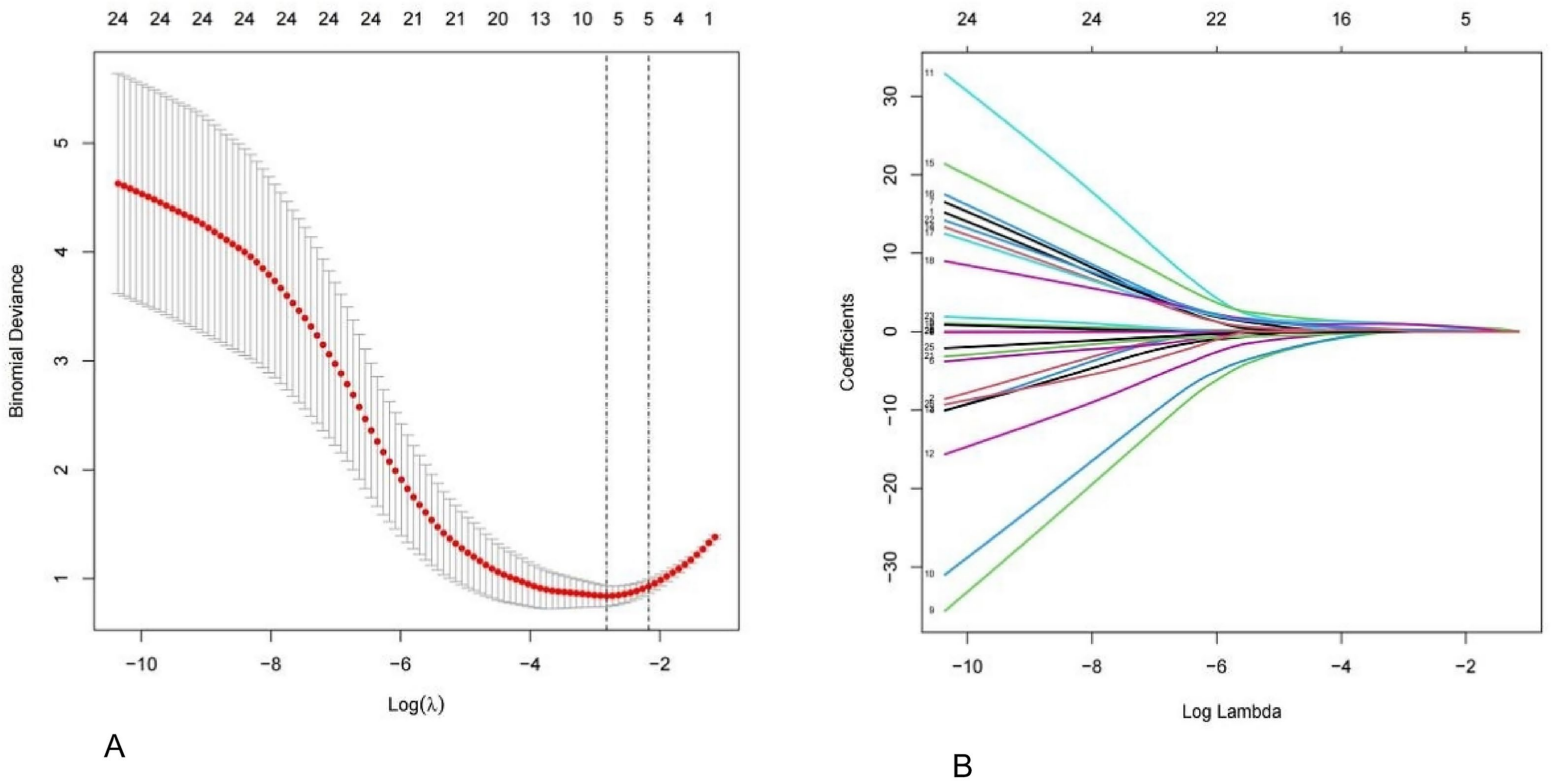
Feature variable assessment process. **(A)** Feature selection based on the LASSO algorithm. The partial likelihood deviance (binomial deviance) curve was plotted vs. log (*λ*). The dotted vertical lines represent the optimal predictors using the minimum criteria (λ.min) and the minimum criteria (λ.1 se). **(B)** A total of seven clinical features were selected based on the coefficients (λ.1 se) under the LASSO algorithm.

The detailed hyperparameter grids and final optimal values for the three models (logistic regression, penalized logistic regression, and XGBoost) are provided in Supplementary Table S1. The original analysis comparing three algorithms was exploratory; to reduce the risk of overfitting and multiple comparison bias, only three representative models are reported in the main manuscript. The results for the other five algorithms are available from the corresponding author upon reasonable request. The detailed hyperparameter grids and final optimal values for all three models are provided in Supplementary Table S1, which is included in the supplementary materials file accompanying this submission (Figure 1).

### 2.7 Model evaluation and interpretability

**Figure 2:**
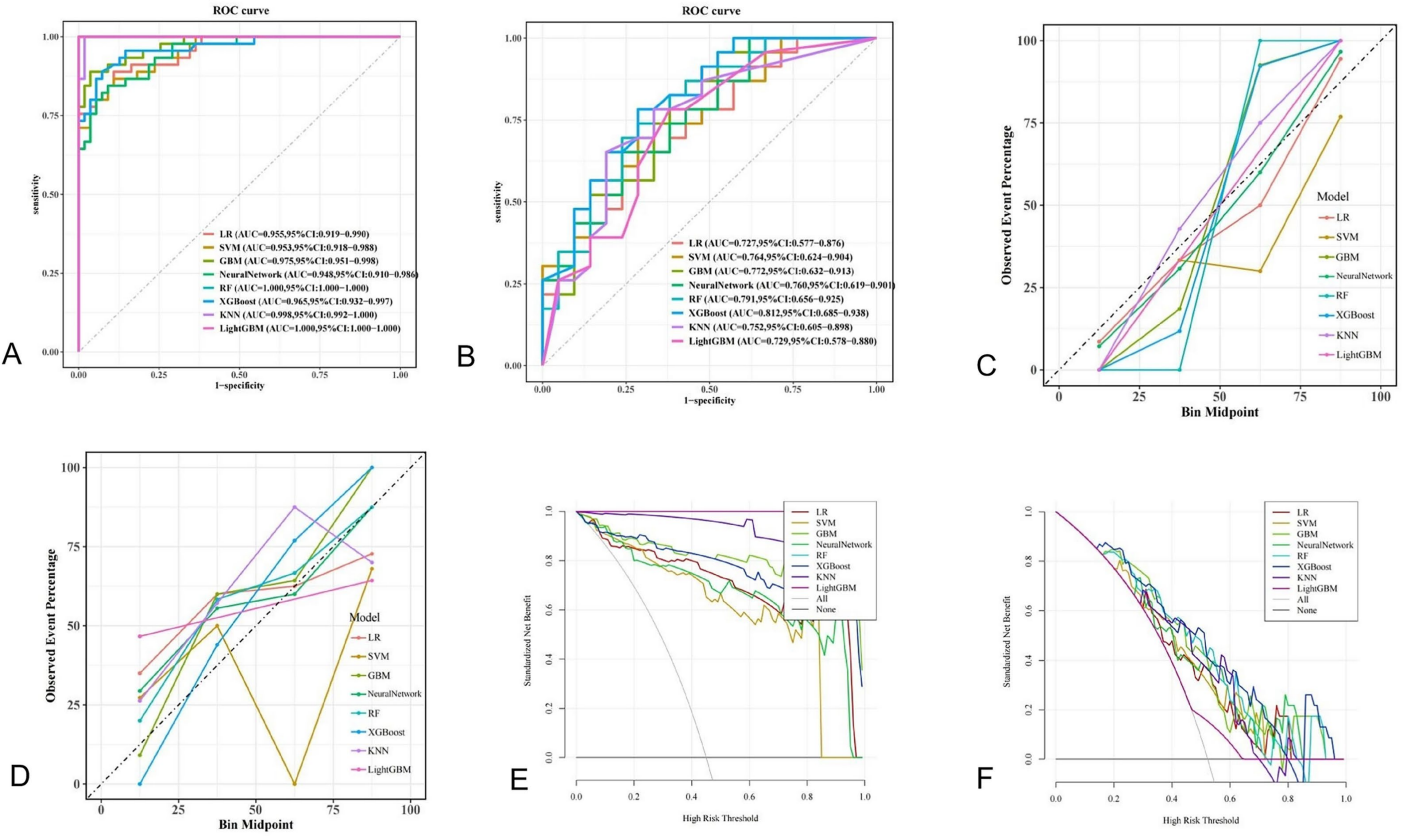
Bootstrap internal validation results for three pre-specified models (LR, penalized logistic regression, and XGBoost). **(A)** Bootstrap-averaged ROC curves with 95% confidence bands (based on 1,000 iterations). **(B)** Bootstrap-aggregated calibration curve for the XGBoost model. The solid line represents the mean predicted probability versus the observed proportion of malnutrition risk, averaged across 1,000 bootstrap iterations. The shaded area represents the 95% confidence interval of the predicted probabilities derived from the bootstrap distribution. The dashed diagonal line indicates perfect calibration. **(C)** Decision curve analysis showing net benefit across threshold probabilities for a random subset of 100 bootstrap iterations (light gray lines) and the mean net benefit (solid colored lines). **(D)** Distribution of C-index values across 1,000 bootstrap iterations for each model, with boxplots indicating median and interquartile range. (Decision curve analysis results, previously referred to as **(E,F)**, are now presented in Supplementary Figure S2 to maintain focus on primary performance metrics in the main figure).

This study evaluated model performance using the bootstrap internal validation approach across three domains: discrimination, calibration, and clinical utility. Discrimination was assessed using the area under the receiver operating characteristic curve and the concordance index, as well as accuracy, sensitivity, specificity, precision, and F1-score, with bootstrap-derived mean, standard deviation, and 95% confidence intervals reported for all metrics. Calibration was examined using calibration curves and two quantitative calibration metrics: the calibration intercept (assessing whether predicted probabilities are systematically biased toward overestimation or underestimation) and the calibration slope (evaluating the spread of predicted risks), along with the Brier score as an overall measure of probabilistic prediction error. For each bootstrap iteration, the calibration intercept and slope were estimated by regressing the observed outcome against the predicted probabilities; their means and 95% confidence intervals were then reported. Calibration curves were generated for each bootstrap iteration and summarized by plotting the mean predicted probability against the observed proportion, with 95% confidence bands derived from the bootstrap distribution (Figure 2B). Perfect calibration corresponds to an intercept of 0 and a slope of 1. An intercept significantly different from 0 suggests systematic overprediction (intercept < 0) or underprediction (intercept > 0), while a slope significantly different from 1 indicates that predicted risks are either too extreme (slope < 1) or too conservative (slope > 1). Clinical utility was evaluated using decision curve analysis, which estimates net benefit across a range of decision thresholds; a model was considered clinically useful when its decision curve exceeded the reference strategies of treating all patients and treating none. Given the bootstrap resampling framework, decision curves were plotted for a representative subset of bootstrap iterations to illustrate variability (17). The optimal model was selected based on an integrated assessment of discrimination, calibration, and clinical utility, with particular attention to the stability of performance estimates as reflected by bootstrap confidence intervals.

To improve model interpretability, we applied Shapley Additive exPlanations (SHAP), a game theory-based approach, to the best-performing model fitted on the original full dataset after confirming acceptable bootstrap performance. SHAP quantifies the contribution of each feature to individual predictions by computing Shapley values, enabling both global and local interpretations of the model’s decisions. Visualization tools were used to illustrate the impacts of features, demonstrating that SHAP effectively enhances model transparency and provides a theoretical basis for model refinement and clinical implementation.

### 2.8 Online interactive web-based computational tool (exploratory prototype)

Using the finalized assessment model, this study utilized the Shiny package in R to develop an online, interactive tool. This tool is intended for hypothesis generation and research purposes only. It has not undergone external validation and is not ready for clinical deployment. Users enter predictive variables, and the tool provides real-time, individual risk predictions in probability form. A clear disclaimer to this effect is prominently displayed on the tool’s interface and in Section 3.5.

### 2.9 TRIPOD AI statement

This study strictly adheres to the specifications of the “Transparent Reporting of a Multivariable Prediction Model for Individual Prognosis or Diagnosis - Artificial Intelligence Extension” (TRIPOD-AI) guidelines during the development, validation, evaluation, and reporting of machine learning prediction models to ensure transparency in the research process, methodological rigor, and reproducibility of results (18).

## 3 Results

### 3.1 Baseline characteristics

In this study, 149 questionnaires were distributed to the Department of Nephrology at Beijing Luhe Hospital, affiliated with Capital Medical University, and 144 valid questionnaires were returned, yielding an effective response rate of 96.6%. Given the bootstrap internal validation approach employed as the primary analysis, we do not report results from a single 7:3 train-validation split. As demonstrated in previous methodologic work (19), single splits in samples of this size (*n* = 144) produce unstable performance estimates with overly wide confidence intervals. Therefore, all performance results reported below are derived from 1,000 bootstrap iterations.

For descriptive purposes only and strictly to illustrate that the dataset is amenable to random partitioning, we additionally performed a single random split (7:3 ratio, random seed 1,234). The baseline characteristics of this hypothetical training set and validation set are shown in Supplementary Table S1A–C. No significant differences were observed between the two groups for any variable (all *p* > 0.05), confirming the effectiveness of randomization. These tables are provided for illustrative purposes only and do not form the basis for any model performance estimates.

The baseline characteristics of the full cohort (*N* = 144) are presented in Table 1. Malnutrition risk (PG-SGA ≥ 4) was observed in 68 participants (47.2%).

### 3.2 Predictive variable assessment

This study employed the LASSO regression algorithm, combined with L1 regularization, for feature selection. To evaluate the stability of feature selection, we repeated LASSO in each of the 1,000 bootstrap training sets and recorded the frequency with which each candidate predictor was selected. The seven features reported in the final model (age, SPPB, TUG, triceps skinfold thickness, handgrip strength, triglycerides, and albumin) were selected in >80% of bootstrap iterations (age: 94%, SPPB: 91%, TUG: 88%, triceps skinfold thickness: 86%, handgrip strength: 97%, triglycerides: 82%, albumin: 99%). No other candidate predictor exceeded a 50% selection frequency. This high selection frequency supports the robustness of the seven-feature set and indicates that these associations are not merely due to sample-specific fluctuations. The feature selection process is illustrated in Figure 1.

### 3.3 Model construction and performance evaluation

Based on the pre-specified modeling strategy, we developed three models (LR, penalized logistic regression, and XGBoost) using the seven LASSO-selected features. Hyperparameters were tuned using tenfold cross-validation within each bootstrap iteration. Model performance was internally validated using 1,000 bootstrap resamples. Table 2 reports the mean, standard deviation, and 95% confidence intervals for the C-index and Brier score, as well as calibration intercept and calibration slope, derived from the bootstrap distribution. The bootstrapaveraged receiver operating characteristic (ROC) curves for the three prespecified models are presented in Figure 2A.

The bootstrap resampling approach with 1,000 iterations served as the primary performance evaluation method in this study. For additional sensitivity analysis, we also implemented nested five-fold cross-validation (with an inner loop for hyperparameter tuning and an outer loop for performance estimation), which yielded results consistent with the bootstrap estimates [XGBoost C-index: 0.789 (95% CI 0.703–0.862)], confirming the robustness of our findings to the choice of internal validation method. The XGBoost model showed the best overall performance: mean C-index 0.798 (95% CI 0.712–0.871), Brier score 0.208 (0.176–0.244), calibration intercept −0.12 (−0.41 to 0.18), and calibration slope 0.85 (0.62–1.12).

Table 2 presents the complete set of performance metrics with bootstrap-derived 95% confidence intervals for all three models. For XGBoost, the accuracy was 0.745 (95% CI 0.682–0.806), sensitivity 0.721 (95% CI 0.635–0.804), specificity 0.767 (95% CI 0.699–0.832), precision 0.742 (95% CI 0.655–0.825), and F1-score 0.731 (95% CI 0.651–0.808). All metrics showed substantial uncertainty as reflected by the width of the confidence intervals, consistent with the modest sample size.

When comparing the three pre-specified models, XGBoost achieved the highest mean C-index (0.798, 95% CI 0.712–0.871), followed by penalized logistic regression (0.751, 95% CI 0.672–0.819) and standard logistic regression (0.732, 95% CI 0.652–0.802). The improvement in C-index from LR to XGBoost was 0.066 (95% CI 0.021–0.111), indicating a modest but potentially meaningful gain in discriminative performance. However, the overlapping confidence intervals suggest that this difference should be interpreted cautiously given the sample size. For clinical utility, the simpler LR or ridge models may be preferred in settings where the marginal gain from XGBoost does not outweigh the need for model transparency. To further evaluate the added value of the seven predictors, we also constructed a parsimonious reference model using only age and albumin—two routinely available variables. This minimal model achieved a bootstrap-corrected C-index of 0.692 (95% CI 0.618–0.763), which was significantly lower than that of the seven-predictor XGBoost model (difference 0.106, 95% CI 0.047–0.165), supporting the incremental predictive value of the physical function measures.

The 95% CI width for C-index (0.159) indicates substantial uncertainty given the EPV of 9.7. The calibration slope being less than 1 suggests that predicted risks may be slightly overconfident (i.e., too extreme), although the confidence interval includes 1. The intercept near zero indicates no systematic overprediction or underprediction. The distribution of C-index values across the 1000 bootstrap iterations for each model is shown in Figure 2F (boxplots with median and interquartile range).

Figure 2B presents the bootstrap-aggregated calibration curve for the XGBoost model, showing good agreement between predicted and observed probabilities across the range of 0.2 to 0.8, with somewhat wider confidence bands at the extremes (predicted probabilities >0.8) due to fewer observations in this range. The variability of the Cindex across the 1,000 bootstrap iterations is illustrated using boxplots in Figure 2D.

Decision curve analysis based on the bootstrap samples showed that XGBoost provided a positive net benefit across threshold probabilities of 0.2 to 0.7 in the majority of iterations (Figure 2C). To quantify the uncertainty, the median net benefit at a threshold probability of 0.4 was 0.12 (95% bootstrap CI: 0.04 to 0.19). The complete distribution of net benefit across all threshold probabilities is summarized in Figure 2E.

### 3.4 Model interpretability

**Figure 3:**
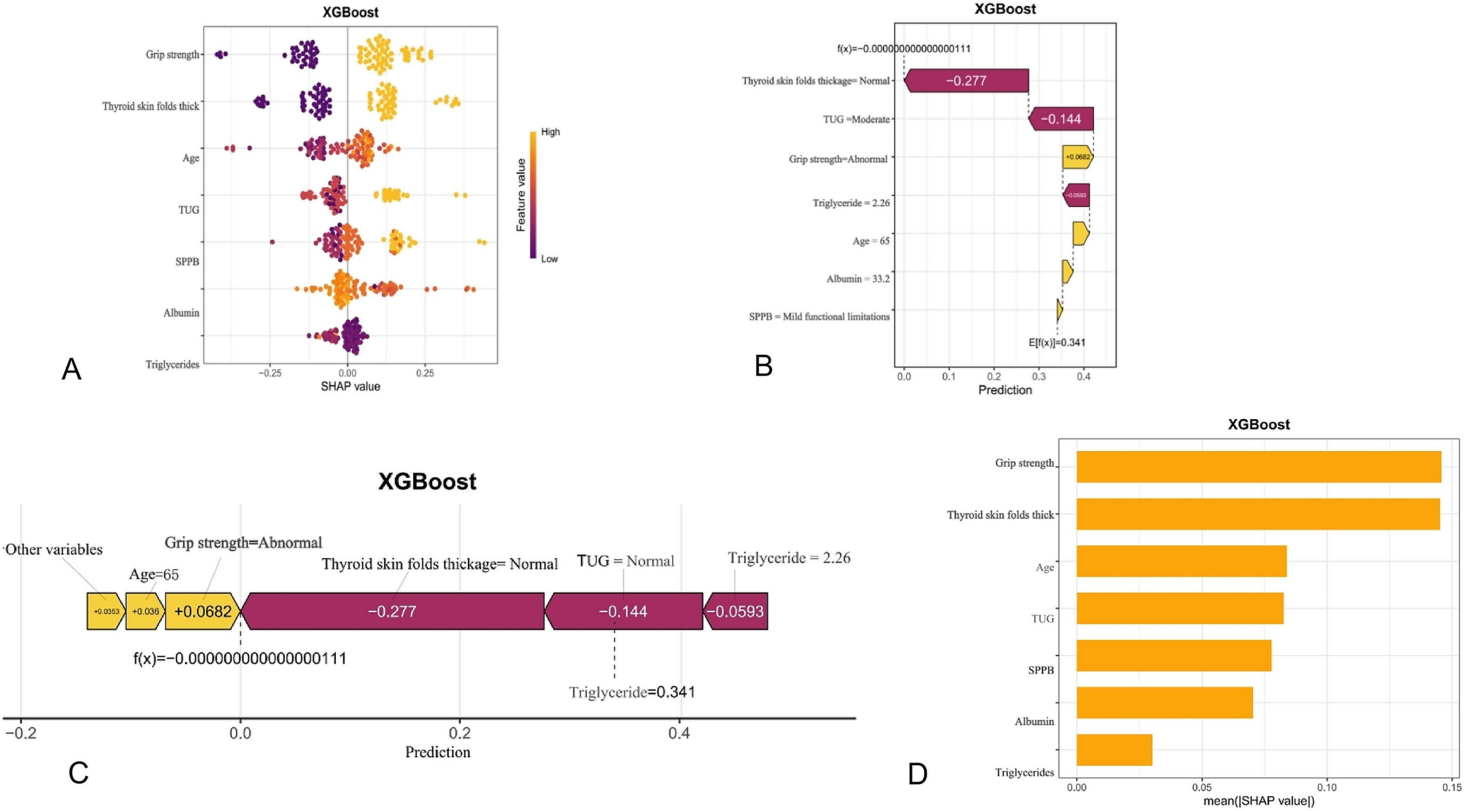
**(A)** Hive plot of the SHA*p* values of the model constructed by the XGBoost algorithm. Vertical coordinates show the importance of the features, sorted in descending order of variable importance, while the variables above are more important to the model. For horizontal positions, the ‘Shap value’ shows whether the effect of this value is related to higher or lower predictions. The color bar label has been changed from “Feature value” to “Feature magnitude” to accurately reflect that the color coding represents the relative magnitude of each feature within its own distribution, rather than absolute values comparable across different features. The color of each SHAP value point indicates whether the observed value is high (yellow) or low (purple). **(B)** The waterfall plot of SHAP values for the model constructed by the XGBoost algorithm. **(C)** SHAP value force plot of the model constructed using the XGBoost algorithm. **(D)** The SHAP variable importance ranking plot of the model constructed using the XGBoost algorithm.

To thoroughly analyze the prediction mechanism and feature contributions of the XGBoost model, this study employs the SHAP method for visual interpretation. Figure 3A presents the SHAP beeswarm plot, where each dot represents the SHAP value of a specific feature for a given sample. The horizontal axis indicates the direction and magnitude of the feature’s impact on the prediction outcome, with negative values reducing the risk probability and positive values increasing it. The vertical axis ranks features by their importance, from highest to lowest. The color of the dots denotes the magnitude of the feature magnitude, where yellow represents high values and purple indicates low values, visually demonstrating the promotive or inhibitory effects of different feature values on the prediction.

Building upon the global feature interpretation provided by the beeswarm plot, this study further integrates SHAP waterfall plots (Figure 3B) and force plots (Figure 3C) to offer local explanations for individual prediction outcomes. The waterfall plot clearly displays the cumulative contribution of each feature to the predicted value for a specific sample, while the force plot dynamically illustrates how each feature shifts the base prediction toward the final output. In these plots, purple denotes lower feature values, and yellow represents higher values, facilitating the identification of the direction and strength of each factor’s influence on the prediction. For example, in one representative case, the model predicted a malnutrition risk probability of 34.1% based on the following continuous input values: age 65 years, SPPB score 7, TUG time 12.5 s, triceps skinfold thickness 12.0 mm, handgrip strength 24.5 kg, serum albumin 33.2 g/L, and triglycerides 2.26 mmol/L.

Together, these visualization methods help reveal the most influential features in the model. Through SHAP analysis, this study identified grip strength, triceps skinfold thickness, age, TUG, SPPB, albumin, and triglycerides as the key variables influencing the model’s predictions (Figure 3D). Among these, grip strength, triceps skinfold thickness, age, TUG, and SPPB exhibited the highest overall predictive contribution.

### 3.5 Online interactive web-based computational tool for construction

**Figure 4:**
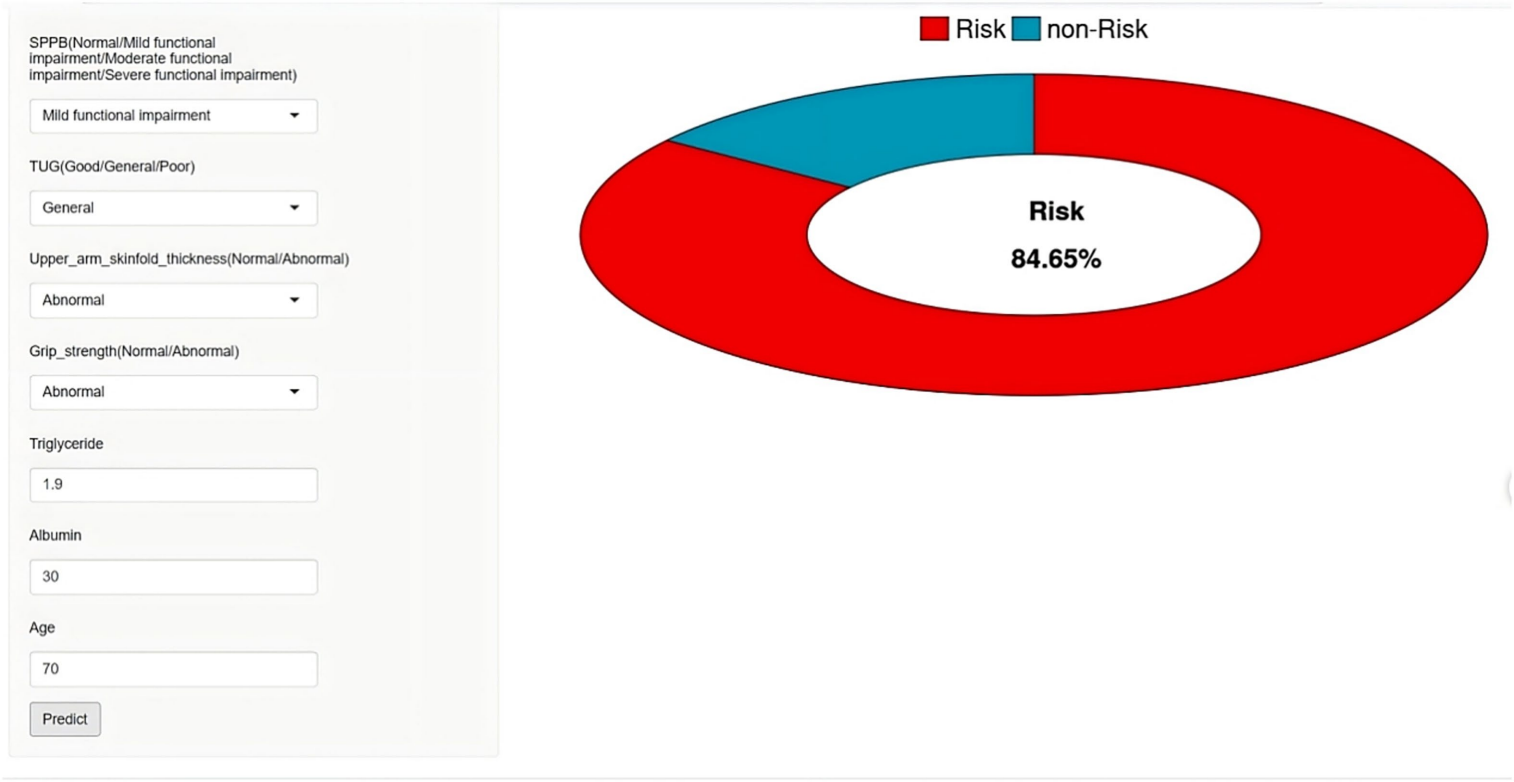
Example prediction result from the online interactive web-based tool. The figure shows a screenshot of the interactive tool’s output for a representative patient (70 years old, SPPB indicating mild functional impairment, moderate TUG test result, poor triceps skinfold thickness, normal grip strength, triglycerides 1.9 mmol/L, albumin 30 g/L), with a predicted malnutrition risk of 84.65%.

To facilitate exploratory analyses and hypothesis generation, we developed an online tool based on the final XGBoost model and deployed it on a web platform.^1^ The tool features a clear interface divided into two sections: an input section for information and a display section for results. IMPORTANT DISCLAIMER: This tool is an exploratory research prototype. It has not undergone external validation and should NOT be used for clinical decision-making. The predictions should be interpreted with extreme caution. The model is intended for hypothesis generation and research purposes only. Clinical assessment of malnutrition risk should always be based on comprehensive evaluation by qualified healthcare professionals. For example, for a 70-year-old patient with an SPPB indicating mild functional impairment, a moderate TUG test result, poor triceps skinfold thickness, normal grip strength, a measured triglyceride level of 1.9 mmol/L, and an albumin level of 30 g/L, the tool predicts their malnutrition risk at 84.65% (Figure 4, which provides an example of the prediction result from the online interactive tool). Users should be aware that the model requires input from specialized assessments (SPPB, TUG, skinfold thickness) and is not intended for rapid bedside screening.

### 3.6 Sensitivity analysis with alternative outcome definition

To assess whether the model’s performance was specific to the chosen PG-SGA threshold of ≥4, we conducted a sensitivity analysis using a stricter outcome definition: PG-SGA ≥ 9 (indicating critical need for urgent symptom management and/or nutritional intervention). Among the 144 participants, 21 (14.6%) met this stricter criterion. Using the same bootstrap internal validation procedure with the stricter outcome, the XGBoost model achieved a mean C-index of 0.756 (95% CI 0.658–0.844), which was lower than the primary analysis (0.798). The reduction in performance is likely due to the smaller number of events (EPV = 3.0 for the stricter outcome) and the more heterogeneous nature of severe malnutrition. These results suggest that the model performs best at detecting moderate malnutrition risk (PG-SGA ≥ 4) and may have limited utility for identifying only the most severe cases. This sensitivity analysis also reinforces the exploratory nature of our findings, as the model’s performance is sensitive to the choice of outcome threshold.

### 3.7 Incremental value of physical function measures

To assess whether the inclusion of physical function measures (SPPB, TUG, handgrip strength) provides additional predictive value beyond routinely available laboratory and demographic variables, we constructed three nested models and compared their bootstrap-corrected performance:


Model1(minimal):age+albumin only

Model2(laboratory+demographics):age+albumin+triglycerides

$$\begin{array}{l} \text{Model}\;3\;(\text{simple physical}+\mathrm{lab}):\text{albumin}+\text{handgrip strength}+\\ \text{triceps skinfold thickness} \end{array}$$

$$\begin{array}{l} \text{Model}\;4\;(\text{full model}):\mathrm{all}\;\text{seven predictors including SPPB},\\ \mathrm{TUG},\text{triceps skinfold thickness},\text{and handgrip strength} \end{array}$$

The C-index values were: Model 1: 0.692 (95% CI 0.618–0.763); Model 2: 0.724 (95% CI 0.648–0.796); Model 3: 0.762 (95% CI 0.685--0.834); Model 4 (full XGBoost): 0.798 (95% CI 0.712--0.871). The improvement from Model 3 to Model 4 (ΔC-index = 0.036, 95% CI 0.012--0.062) further supports the incremental, albeit modest, contribution of the more comprehensive physical function tests (SPPB, TUG) over simpler bedside measures alone.

## 4 Discussion

Malnutrition is an independent risk factor for mortality in PD patients. Furthermore, PD treatment itself may lead to reduced nutritional intake, enhanced protein catabolism, decreased synthesis, and increased loss, thereby adversely affecting patients’ nutritional status (20). In light of these challenges, regular nutritional assessments can not only improve patients’ nutritional status but also reduce complication rates and mortality, serving as a critical component of comprehensive PD patient management (21). Among the various nutritional assessment tools, the PG-SGA score demonstrates high correlation, validity, and practicality. This makes it the preferred tool for nutritional assessment in patients with PD. (22)

Consistent with prior literature, our data confirmed that older age (23, 24), lower handgrip strength (25), reduced triceps skinfold thickness (26), poorer SPPB and TUG performance (27, 28), hypoalbuminemia (29, 30), and lower triglycerides (19, 31–33) were each associated with malnutrition risk in PD patients. The observed association between lower triglyceride levels and malnutrition risk is consistent with findings in other chronic disease populations, where it may reflect depleted energy stores (34). However, these associations are well-established and do not constitute the primary contribution of the present study. Therefore, rather than reiterating these known relationships, the discussion below focuses on three core issues that are directly relevant to prediction model research: (1) interpretation of the model’s performance characteristics after rigorous internal validation; (2) quantitative comparison with existing nutritional assessment tools and simpler predictor combinations; and (3) a practical trade-off analysis of clinical feasibility, balancing measurement burden against predictive gain.

### 4.1 Interpretation of model performance

After bootstrap internal validation (1,000 iterations), the XGBoost model achieved a mean C-index of 0.798 (95% CI 0.712–0.871). In clinical terms, this level of discrimination indicates that when randomly pairing one malnourished patient with one well-nourished patient, the model would correctly assign a higher risk probability to the malnourished individual in approximately 80% of such pairs — a moderate but not excellent ability. The 95% confidence interval (width 0.159) reflects substantial uncertainty due to the modest sample size (EPV = 9.7), meaning the true discriminative performance could range from only fair (C-index ~0.71) to good (~0.87) in different samples from the same population. The calibration intercept of −0.12 (95% CI − 0.41 to 0.18) suggests no systematic overprediction or underprediction at the group level. However, the calibration slope of 0.85 (95% CI 0.62–1.12), while including 1, leans below unity — a pattern that typically indicates overconfidence in extreme predictions (i.e., predicted probabilities near 0 or 1 are too extreme relative to observed frequencies). Clinicians using this model should therefore interpret predicted probabilities with caution, especially at the tails of the risk distribution. The conventional single 7:3 split produced a C-index of 0.812 (95% CI 0.684–0.940) — a much wider interval with a lower bound barely above chance — confirming that single-split validation is overly optimistic and unstable for this sample size. These performance estimates remain exploratory and should not be considered definitive evidence of readiness for clinical deployment.

### 4.2 Quantitative comparison with existing tools and simpler models

A critical question is whether the seven-predictor XGBoost model offers any tangible advantage over simpler predictor combinations. Our comparison showed that the XGBoost model (C-index 0.798) outperformed both standard logistic regression (0.732) and a minimal age-albumin model (0.692). The incremental C-index gain of 0.066 (95% CI 0.021–0.111) over LR suggests that the nonlinear relationships captured by XGBoost provide modest but non-negligible improvements in discriminating malnutrition risk. However, the clinical significance of this gain depends on the specific decision context. For settings where interpretability is paramount (e.g., explaining risk to patients or guiding simple nutritional counseling), the logistic regression model with the same seven predictors represents a reasonable and more transparent alternative.

A critical benchmark for nutritional assessment in dialysis is the Malnutrition Inflammation Score (MIS). We acknowledge that the lack of MIS data in our study prevents a direct, quantitative comparison, which is a major limitation. The MIS, which integrates nutritional and inflammatory parameters, has been validated against clinical outcomes in PD populations and would serve as a natural comparator. Future external validation studies should prioritize collecting MIS alongside the PG-SGA and the predictors used in our model to determine whether the machine learning approach offers any advantage (e.g., in terms of discrimination, calibration, or clinical utility) over this established, though more complex, scoring system. It is recommended that future external validation studies prioritize the collection of MIS data to enable head-to-head performance comparisons between the machine learning model and the MIS.

### 4.3 Clinical feasibility and incremental value of physical function measures

Our nested model comparison demonstrated that adding physical function measures (SPPB, TUG, handgrip strength) to a laboratory-only model (age, albumin, triglycerides) improved the C-index from 0.724 to 0.798 (*Δ* = 0.074). This improvement translates into a moderate gain in discriminative ability, suggesting that physical function captures nutritional risk dimensions not fully reflected in laboratory tests. However, this gain comes at a cost: SPPB, TUG, and skinfold measurements require specialized training, dedicated equipment, and approximately 10–15 min per patient. In a typical busy dialysis unit, universal administration of these tests may not be feasible. A more efficient approach would be a two-stage process: first, use ultra-brief screening tools (e.g., body weight trend over the past 3 months, a single-question appetite assessment such as “How is your appetite?”, or the Short Nutritional Assessment Questionnaire) to identify a higher-risk subgroup (e.g., 20–30% of the PD population). Second, apply the full machine learning model — including SPPB, TUG, and skinfold measurements — only to these positively screened patients for detailed nutritional phenotyping. This staged strategy would preserve most of the predictive gain while reducing measurement burden by 70–80%. Future implementation studies should formally evaluate the net benefit of such a two-stage approach using decision curve analysis with realistic estimates of time costs. Until then, the model should not be promoted as a first-line universal screening tool but rather as a detailed assessment aid for selected high-risk patients. Our nested model comparison provides empirical support for this approach: the full model’s performance advantage over the laboratory-only model (ΔC-index 0.074) suggests that physical function measures contribute non-trivial predictive information, justifying their use in selected high-risk patients.

A fundamental conceptual issue must be re-emphasized. The outcome (PG-SGA ≥ 4) and several predictors (handgrip strength, triceps skinfold thickness, and some physical function measures) reflect overlapping nutritional constructs. Handgrip strength and skinfold thickness directly contribute to PG-SGA scoring. Therefore, model performance may partly reflect circularity rather than genuine independent prediction. The model should therefore be viewed as an alternative representation of the PG-SGA rather than a tool that provides information beyond that captured by the PG-SGA itself. This does not invalidate the model but fundamentally redefines its intended use: it identifies factors already consistent with the PG-SGA definition, not future or independent risk. In exploratory research, such circularity is acceptable as long as it is explicitly acknowledged and the model is not promoted as providing independent prognostic value.

This study has several additional limitations. First, the sample size and outcome events were modest (EPV = 9.7, below the recommended minimum of 10). Despite using 1,000 bootstrap iterations and pre-specifying only three algorithms, the bootstrap-corrected performance estimates still showed considerable uncertainty, and the optimism observed in the single-split validation was not entirely eliminated (35). External validation in larger independent cohorts is strictly required. Second, the cross-sectional design precludes evaluation of temporal changes, causality, or long-term outcomes. Third, convenience sampling from a single center introduces selection bias and limits representativeness. Finally, variations in measurement protocols across institutions (e.g., SPPB, TUG, skinfold technique) may affect external transferability (14). Model updating or recalibration may be necessary before broader implementation.

The present model should not be interpreted as providing independent prognostic information beyond the PG-SGA itself, given the conceptual overlap between several predictors and the outcome. The interpretability analysis using SHAP and the online tool may support hypothesis generation and individualized nutritional assessment within similar clinical settings, but external validation in large, independent cohorts is mandatory before any clinical deployment. Pending such validation, and particularly pending direct comparisons with simpler tools (e.g., albumin-age model, MIS) and formal cost–benefit analyses of the two-stage screening strategy, the present results should be considered exploratory.

## 5 Conclusion

This study explored the feasibility of a machine learning model to associate with current PG-SGA-defined malnutrition risk in a single-center PD cohort. After limiting compared algorithms to three and using bootstrap internal validation, the XGBoost model showed moderate discrimination (C-index 0.798, 95% CI 0.712–0.871) and acceptable calibration (intercept −0.12, slope 0.85) in the development cohort. However, these performance estimates remain uncertain due to the modest sample size (EPV 9.7) and the single-center cross-sectional design. The model should not be interpreted as providing independent prognostic information beyond the PG-SGA itself, given the conceptual overlap between several predictors and the outcome. The model is best understood as an alternative empirical representation of the PG-SGA construct. The interpretability analysis using SHAP and the online tool may support hypothesis generation and individualized nutritional assessment within similar clinical settings, but external validation in large, independent cohorts is mandatory before any clinical deployment. The online tool is provided as an exploratory research prototype and is not ready for clinical use. Pending such validation, and particularly pending direct comparisons with simpler tools and formal cost–benefit analyses, the present results should be considered hypothesis-generating only.

## Data Availability

The original contributions presented in the study are included in the article/supplementary material, further inquiries can be directed to the corresponding author/s.

## Footnotes

1
https://predictionmode.shinyapps.io/make_web

## References

[1] Li PK Chow KM Van de Luijtgaarden MW Johnson DW Jager KJ Mehrotra R . Changes in the worldwide epidemiology of peritoneal dialysis. Nat Rev Nephrol. (2017) 13:90–103. doi: 10.1038/nrneph.2016.181 28029154

[2] Ikizler TA Burrowes JD Byham-Gray LD Campbell KL Carrero JJ Chan W . KDOQI clinical practice guideline for nutrition in CKD: 2020 update. Am J Kidney Dis. (2020) 76:S1–1S107. doi: 10.1053/j.ajkd.2020.05.006 32829751

[3] Rashid I Bashir A Tiwari P D'Cruz S Jaswal S. Estimates of malnutrition associated with chronic kidney disease patients globally and its contrast with India: an evidence-based systematic review and meta-analysis. Clin Epidemiol Glob Health. (2021) 12:100855. doi: 10.1016/j.cegh.2021.100855

[4] Canada-USA (CANUSA) Peritoneal Dialysis Study Group. Adequacy of dialysis and nutrition in continuous peritoneal dialysis: association with clinical outcomes. Canada-USA (CANUSA) Peritoneal Dialysis Study Group. J Am Soc Nephrol. (1996) 7:198–207. doi: 10.1681/ASN.V72198 8785388

[5] Yang Y Xu Y Zhang P Zhou H Yang M Xiang L. Predictive value of objective nutritional indices in technique failure in peritoneal Dialysis patients. J Ren Nutr. (2022) 32:605–12. doi: 10.1053/j.jrn.2021.09.005, 34776339

[6] Kittiskulnam P Chuengsaman P Kanjanabuch T Katesomboon S Tungsanga S Tiskajornsiri K . Protein-energy wasting and mortality risk prediction among peritoneal Dialysis patients. J Ren Nutr. (2021) 31:679–86. doi: 10.1053/j.jrn.2020.11.007, 33642190

[7] Paniagua R Amato D Correa-Rotter R Ramos A Vonesh EF Mujais SK. Correlation between peritoneal equilibration test and dialysis adequacy and transport test, for peritoneal transport type characterization. Perit Dial Int. (2000) 20:53–9. doi: 10.1177/089686080002000110, 10716584

[8] Cai G Zhang Y Pan M Zhou S Xiang X Ying J. Explainable machine learning models for predicting of protein-energy wasting in patients on maintenance haemodialysis. BMC Nephrol. (2025) 26:562. doi: 10.1186/s12882-025-04476-7, 41087938 PMC12523148

[9] Riley RD Ensor J Snell K Harrell FE Martin GP Reitsma JB . Calculating the sample size required for developing a clinical prediction model. BMJ. (2020) 368:m441. doi: 10.1136/bmj.m441 32188600

[10] Bauer J Capra S Ferguson M. Use of the scored patient-generated subjective global assessment (PG-SGA) as a nutrition assessment tool in patients with cancer. Eur J Clin Nutr. (2002) 56:779–85. doi: 10.1038/sj.ejcn.1601412, 12122555

[11] Kempen GI Yardley L van Haastregt JC Zijlstra GA Beyer N Hauer K . The short FES-I: a shortened version of the falls efficacy scale-international to assess fear of falling. Age Ageing. (2008) 37:45–50. doi: 10.1093/aging/afm157 18032400

[12] Woo J Yu R Wong M Yeung F Wong M Lum C. Frailty screening in the community using the FRAIL scale. J Am Med Dir Assoc. (2015) 16:412–9. doi: 10.1016/j.jamda.2015.01.087, 25732832

[13] Ramírez-Vélez R Pérez-Sousa MA Venegas-Sanabria LC Cano-Gutierrez CA Hernández-Quiñonez PA Rincón-Pabón D . Normative values for the short physical performance battery (SPPB) and their association with anthropometric variables in older Colombian adults. The SABE study, 2015. Front Med (Lausanne). (2020) 7:52. doi: 10.3389/fmed.2020.00052, 32154258 PMC7044127

[14] Podsiadlo D Richardson S. The timed “up and go”: a test of basic functional mobility for frail elderly persons. J Am Geriatr Soc. (1991) 39:142–8. doi: 10.1111/j.1532-5415.1991.tb01616.x 1991946

[15] Sandhu R Lee TH. Incorporating handgrip strength examination into dietetic practice: a quality improvement project. Nutr Clin Pract. (2023) 38:904–13. doi: 10.1002/ncp.10972, 36847695

[16] Yang N He LY Li ZY Yang YC Ping F Xu LL . Triceps skinfold thickness trajectories and the risk of all-cause mortality: a prospective cohort study. World J Clin Cases. (2024) 12:2568–77. doi: 10.12998/wjcc.v12.i15.2568, 38817233 PMC11135450

[17] Hanley JA McNeil BJ. The meaning and use of the area under a receiver operating characteristic (ROC) curve. Radiology. (1982) 143:29–36. doi: 10.1148/radiology.143.1.7063747, 7063747

[18] Collins GS Moons K Dhiman P Riley RD Beam AL Van Calster B . TRIPOD+AI statement: updated guidance for reporting clinical prediction models that use regression or machine learning methods. BMJ. (2024) 385:e078378. doi: 10.1136/bmj-2023-078378, 38626948 PMC11019967

[19] Miller M. Is triglyceride therapy worth the effort. Cleve Clin J Med. (2015) 82:162–6. doi: 10.3949/ccjm.82a.13157, 25932741

[20] Huang H Wang Q Luo Y Tang Z Liu F Zhang R . Validity and applicability of the global leadership initiative on malnutrition criteria in nondialysis patients with chronic kidney disease. Front Nutr. (2024) 11:1340153. doi: 10.3389/fnut.2024.1340153, 38362100 PMC10867223

[21] Kiebalo T Holotka J Habura I Pawlaczyk K. Nutritional status in peritoneal Dialysis: nutritional guidelines, adequacy and the Management of Malnutrition. Nutrients. (2020) 12:12. doi: 10.3390/nu12061715, 32521626 PMC7352713

[22] Chen X Liu X Ji W Zhao Y He Y Liu Y . The PG-SGA outperforms the NRS 2002 for nutritional risk screening in cancer patients: a retrospective study from China. Front Nutr. (2023) 10:1272420. doi: 10.3389/fnut.2023.1272420, 38075213 PMC10702952

[23] Johansson L. Nutrition in older adults on peritoneal Dialysis. Perit Dial Int. (2015) 35:655–8. doi: 10.3747/pdi.2014.00343, 26702008 PMC4689469

[24] Norman K Haß U Pirlich M. Malnutrition in older adults-recent advances and remaining challenges. Nutrients. (2021) 13:13. doi: 10.3390/nu13082764, 34444924 PMC8399049

[25] Wang AY Sea MM Ho ZS Lui SF Li PK Woo J. Evaluation of handgrip strength as a nutritional marker and prognostic indicator in peritoneal dialysis patients. Am J Clin Nutr. (2005) 81:79–86. doi: 10.1093/ajcn/81.1.79, 15640464

[26] Vaquero-Cristóbal R Catarina-Moreira A Esparza-Ros F Barrigas C Albaladejo-Saura M Vieira F. Skinfolds compressibility and digital caliper's time response in skinfold measurement in male and female young adults. J Int Soc Sports Nutr. (2023) 20:2265888. doi: 10.1080/15502783.2023.2265888, 37794782 PMC10557569

[27] Beretta MV De Paula TP Da Costa Rodrigues T Steemburgo T. Prolonged hospitalization and 1-year mortality are associated with sarcopenia and malnutrition in older patients with type 2 diabetes: a prospective cohort study. Diabetes Res Clin Pract. (2024) 207:111063. doi: 10.1016/j.diabres.2023.111063, 38110120

[28] Ramsey KA Meskers C Trappenburg MC Verlaan S Reijnierse EM Whittaker AC . Malnutrition is associated with dynamic physical performance. Aging Clin Exp Res. (2020) 32:1085–92. doi: 10.1007/s40520-019-01295-3, 31429000 PMC7260152

[29] Dumler F. Hypoalbuminemia is a marker of overhydration in chronic maintenance patients on dialysis. ASAIO J. (2003) 49:282–6. doi: 10.1097/01.mat.0000065465.52748.bb, 12790376

[30] Yuan D Wang XQ Shao F Zhou JJ Li ZX. Study on the occurrence and influencing factors of gastrointestinal symptoms in hemodialysis patients with uremia. World J Gastrointest Surg. (2024) 16:2157–66. doi: 10.4240/wjgs.v16.i7.2157, 39087119 PMC11287689

[31] Awad D Cao P Pulliam TL Spradlin M Subramani E Tellman TV . Adipose triglyceride lipase is a therapeutic target in advanced prostate cancer that promotes metabolic plasticity. Cancer Res. (2024) 84:703–24. doi: 10.1158/0008-5472.CAN-23-0555, 38038968 PMC10939928

[32] Hunter PM Hegele RA. Functional foods and dietary supplements for the management of dyslipidemia. Nat Rev Endocrinol. (2017) 13:278–88. doi: 10.1038/nrendo.2016.210, 28133369

[33] Lazarow H Singer R Compher C Gilmar C Kucharczuk CR Mangan P . Effect of malnutrition-driven nutritional support protocol on clinical outcomes in autologous stem cell transplantation patients. Support Care Cancer. (2021) 29:997–1003. doi: 10.1007/s00520-020-05571-1, 32556621

[34] Yu JM Yang M Xu HX Li W Fu ZM Lin Y . Investigation on Nutrition Status and Clinical Outcome of Common Cancers (INSCOC) Group. Association Between Serum C-Reactive Protein Concentration and Nutritional Status of Malignant Tumor Patients. Nutr Cancer. (2019) 71:240–245. doi: 10.1080/01635581.2018.1524019 30450976

[35] Steyerberg EW Harrell FE. Prediction models need appropriate internal, internal-external, and external validation. J Clin Epidemiol. (2016) 69:245–7. doi: 10.1016/j.jclinepi.2015.04.005, 25981519 PMC5578404

