# Supplementary material for: Applying machine learning to associate clinical factors with malnutrition risk in peritoneal dialysis patients: an internally validated interpretable model
Source: Front Nutr. 2026 Jun 18;13:1856219. doi: 10.3389/fnut.2026.1856219 (PMC13322838; doi:10.3389/fnut.2026.1856219)
Supplement: Supplementary file 1 [file Supplementary_file_1.docx]

**Supplementary Table S1A.** Comparison of demographic and clinical characteristics between the hypothetical training set and validation set derived from a single random 7:3 split

| Variables | Training set  (n = 100) | Validation set  (n = 44) | *P* |
| --- | --- | --- | --- |
|  |  |  |  |
| Age (years), Mean±SD | 60.15 ± 13.12 | 59.95 ± 10.34 | 0.924 |
| Male , n(%) | 64 (64.00) | 29 (65.91) | 0.825 |
| Smoking history, n(%) | 50 (50.00) | 21 (47.73) | 0.802 |
| Drinking History, n(%) | 30 (30.00) | 12 (27.27) | 0.740 |
| Education ≥ junior high school, n(%) | 68 (68.00) | 31 (70.45) | 0.770 |
| Diabetes mellitus, n(%) | 33 (33.00) | 17 (38.64) | 0.513 |
| Hypertension, n(%) | 24 (24.00) | 14 (31.82) | 0.327 |
| Nephritis, n(%) | 23 (23.00) | 9 (20.45) | 0.735 |
| Medical Insurance Type (non‑agricultural), n(%) | 54 (54.00) | 26 (59.09) | 0.571 |

Note: P values from chi‑square test for categorical variables and independent t‑test for continuous variables. This table is provided for illustrative purposes only to demonstrate that the dataset is amenable to random partitioning and does not form the basis for any model performance estimates.

**Supplementary Table S1B.** Comparison of laboratory indices between the hypothetical training set and validation set derived from a single random 7:3 split

| Variables | Training set  (n = 100) | Validation set  (n = 44) | *P* |
| --- | --- | --- | --- |
|  |  |  |  |
| Hemoglobin(g/L), Mean±SD | 116.86 ± 13.13 | 116.52 ± 13.21 | 0.888 |
| Total cholesterol(mmol/L), Mean±SD | 3.85 ± 0.96 | 4.15 ± 1.14 | 0.096 |
| Hemoglobin (g/L), Mean±SD | 116.86 ± 13.13 | 116.52 ± 13.21 | 0.888 |
| Total cholesterol (mmol/L), Mean±SD | 3.85 ± 0.96 | 4.15 ± 1.14 | 0.096 |
| Potassium (mmol/L), Mean±SD | 4.20 ± 0.80 | 4.26 ± 0.72 | 0.629 |
| Triglycerides (mmol/L), median (IQR) | 1.59 (1.12, 2.33) | 1.40 (1.06, 2.12) | 0.348 |
| Parathyroid hormone (pg/mL), median (IQR) | 151.30 (60.31, 282.27) | 196.00 (138.30, 291.82) | 0.282 |
| Albumin (g/L), median (IQR) | 35.35 (32.60, 38.25) | 35.55 (33.23, 38.32) | 0.755 |
| Phosphorus (mmol/L), median (IQR) | 1.60 (1.33, 1.98) | 1.63 (1.38, 1.90) | 0.786 |

Note: P values from independent t‑test (normal distribution) or Mann‑Whitney U test (non‑normal). This table is provided for illustrative purposes only.

**Supplementary Table S1C.** Comparison of functional and nutritional assessments between the hypothetical training set and validation set derived from a single random 7:3 split

| Variables | Training set  (n = 100) | Validation set  (n = 44) | *P* |
| --- | --- | --- | --- |
|  |  |  |  |
| Fall risk (high), n(%) | 56 (56.00) | 24 (54.55) | 0.871 |
| Frailty status, n(%) |  |  | 0.572 |
| None | 36 (36.00) | 13 (29.55) |  |
| Prefrailty | 45 (45.00) | 24 (54.55) |  |
| Frailty | 19 (19.00) | 7 (15.91) |  |
| SPPB category, n(%) |  |  | 0.606 |
| Normal | 7 (7.00) | 4 (9.09) |  |
| Mild limitation | 29 (29.00) | 16 (36.36) |  |
| Moderate limitation | 39 (39.00) | 17 (38.64) |  |
| Severe limitation | 25 (25.00) | 7 (15.91) |  |
| TUG category, n(%) |  |  | 0.482 |
| Good | 19 (19.00) | 5 (11.36) |  |
| General | 53 (53.00) | 27 (61.36) |  |
| Poor | 28 (28.00) | 12 (27.27) |  |
| Triceps skinfold thickness (abnormal), n(%) | 50 (50.00) | 19 (43.18) | 0.451 |
| Grip strength (abnormal), n(%) | 63 (63.00) | 27 (61.36) | 0.852 |
| Sarcopenia, n(%) | 51 (51.00) | 16 (36.36) | 0.105 |
| PG‑SGA ≥4 (malnutrition risk), n(%) | 45 (45.00) | 23 (52.27) | 0.421 |
| Insufficient dialysis, n(%) | 18 (18.00) | 10 (22.73) | 0.509 |

Note: P values from chi‑square test or Fisher's exact test. This table is provided for illustrative purposes only and does not form the basis for any model performance estimates.


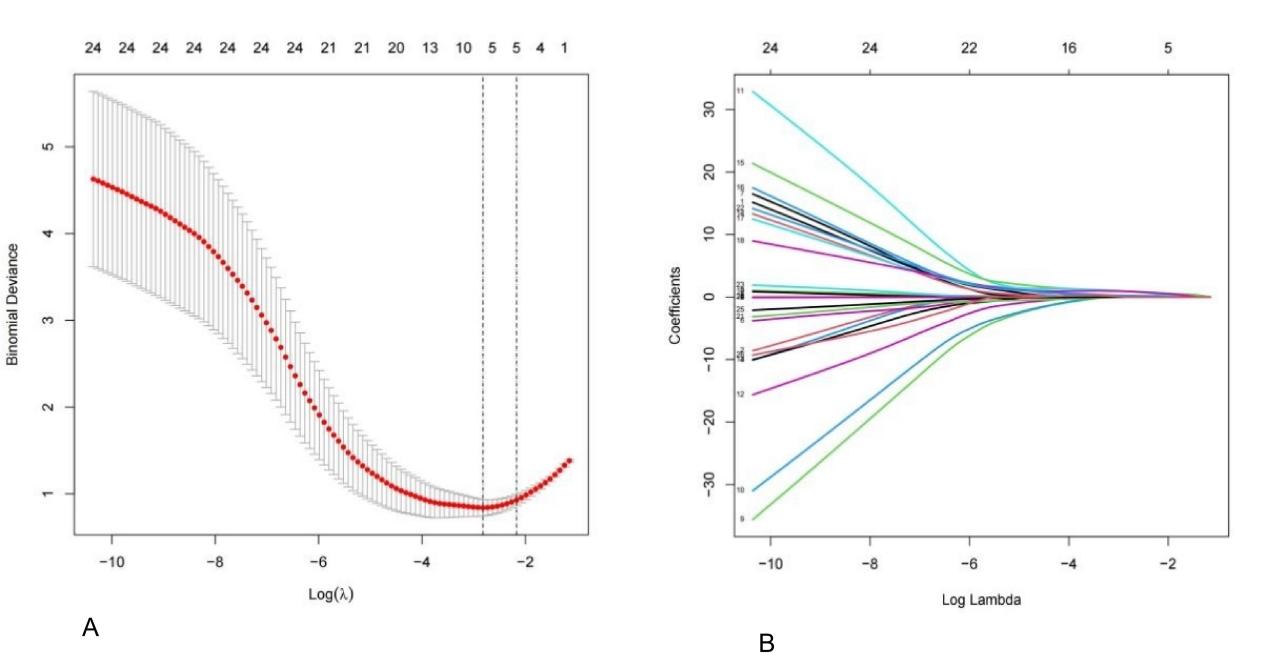


**Figure 1.** Feature variable assessment process. **(A)** Feature selection based on

the LASSO algorithm. The partial likelihood deviance (binomial deviance)

curve was plotted vs. log (λ). The dotted vertical lines represent the optimal

predictors using the minimum criteria (λ.min) and the minimum criteria

(λ.1 se). **(B)** A total of seven clinical features were selected based on the

coefficients (λ.1 se) under the LASSO algorithm.


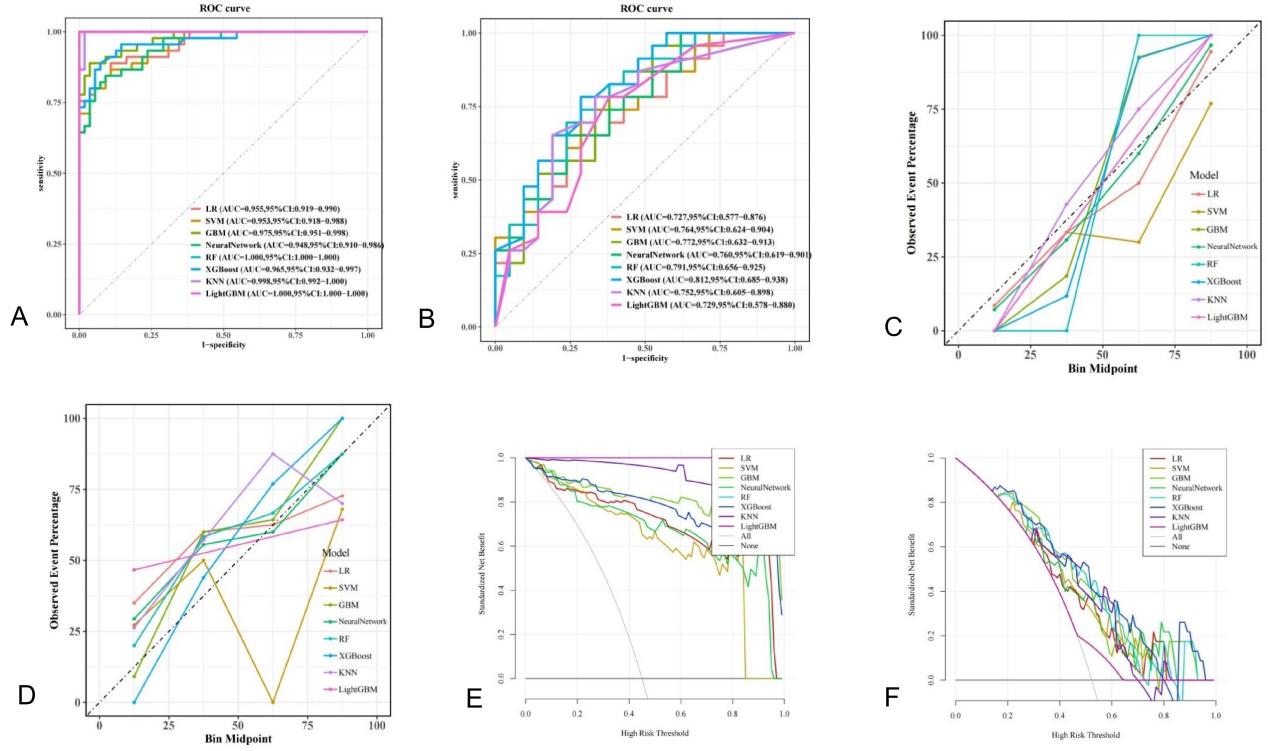


**Figure 2.** Bootstrap internal validation results for three pre‑specified models (LR, penalized logistic regression, and XGBoost). (A) Bootstrap‑averaged ROC curves with 95% confidence bands (based on 1000 iterations). (B) Bootstrap-aggregated calibration curve for the XGBoost model.The solid line represents the mean predicted probability versus the observed proportion of malnutrition risk, averaged across 1000 bootstrap iterations. The shaded area represents the 95% confidence interval of the predicted probabilities derived from the bootstrap distribution. The dashed diagonal line indicates perfect calibration. (C) Decision curve analysis showing net benefit across threshold probabilities for a random subset of 100 bootstrap iterations (light gray lines) and the mean net benefit (solid colored lines). (D) Distribution of C-index values across 1000 bootstrap iterations for each model, with boxplots indicating median and interquartile range. (Note: Decision curve analysis results, previously referred to as Figure 2E-F, are now presented in Supplementary Figure S2 to maintain focus on primary performance metrics in the main figure.)


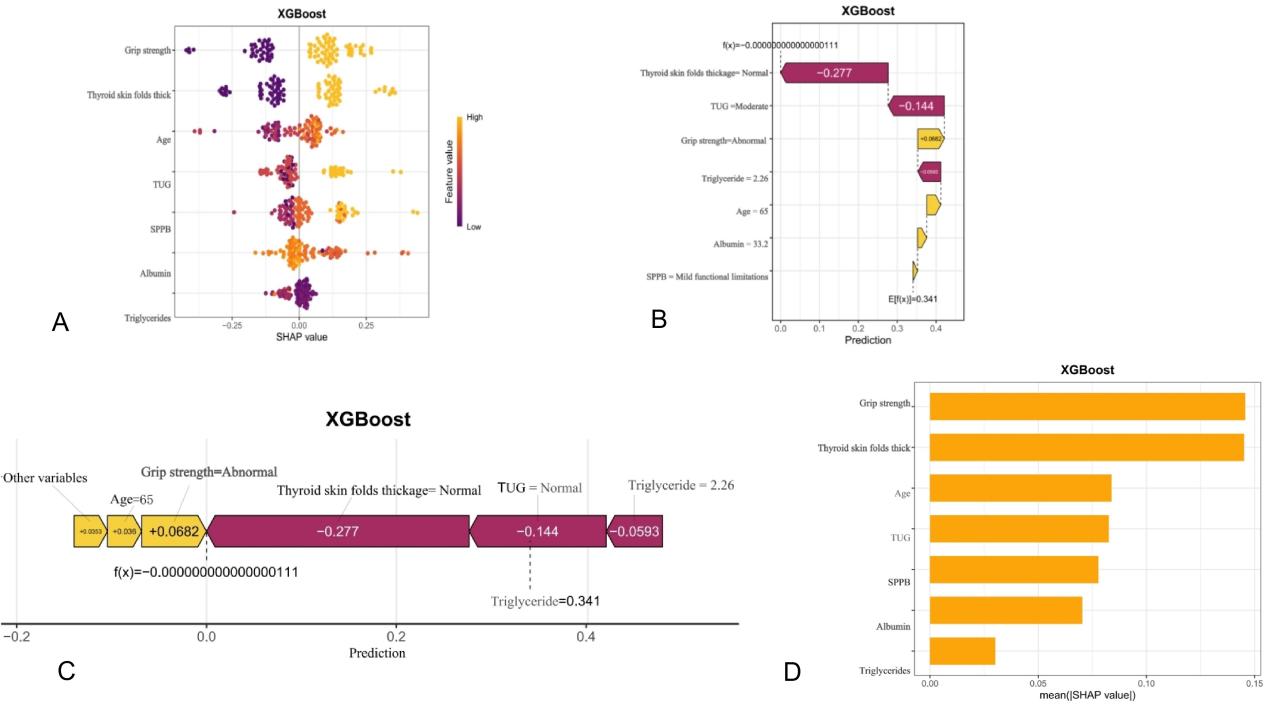


**Figure 3** (A) Hive plot of the SHAP values of the model constructed by the XGBoost algorithm. Vertical coordinates show the importance of the features, sorted in descending order of variable importance, while the variables above are more important to the model. For horizontal positions, the 'Shap value' shows whether the effect of this value is related to higher or lower predictions. The color bar label has been changed from "Feature value" to "Feature magnitude" to accurately reflect that the color coding represents the relative magnitude of each feature within its own distribution, rather than absolute values comparable across different features. The color of each SHAP value point indicates whether the observed value is high (yellow) or low (purple). (B) The Waterfall plot of SHAP values for the model constructed by the XGBoost algorithm. (C) SHAP value force plot of the model constructed using the XGBoost algorithm. (D) The SHAP variable importance ranking plot of the model constructed using the XGBoost algorithm.


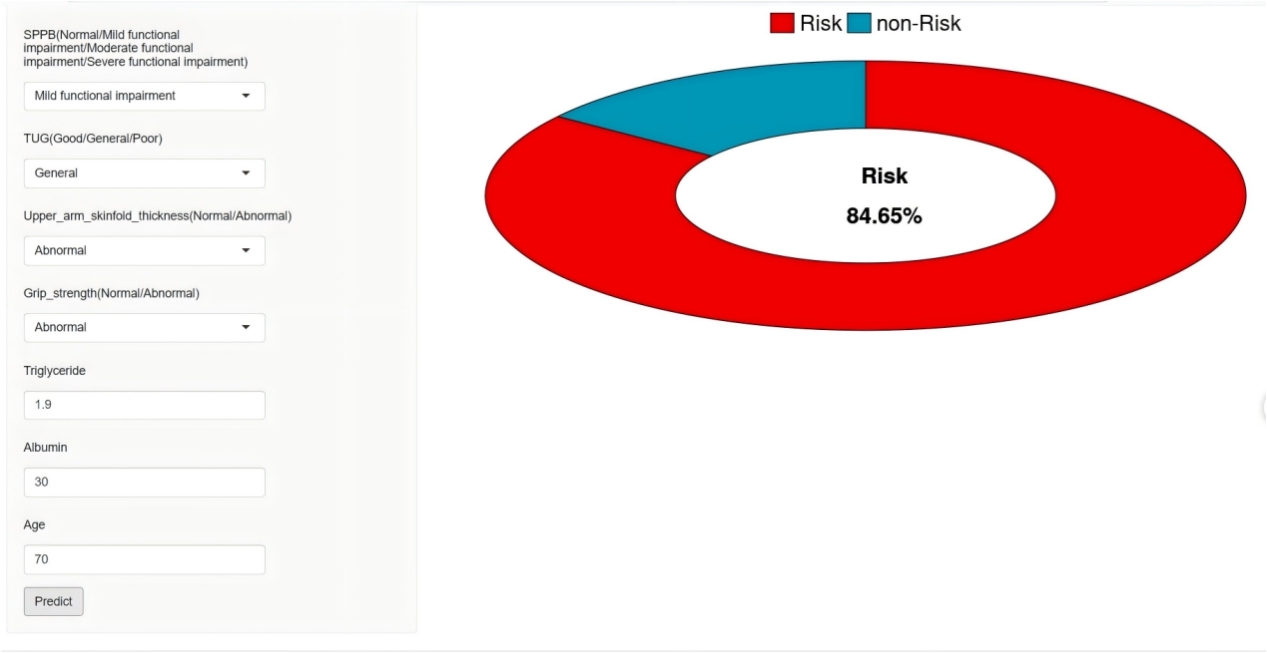


**Figure 4.** Example prediction result from the online interactive web-based tool. The figure shows a screenshot of the interactive tool's output for a representative patient (70 years old, SPPB indicating mild functional impairment, moderate TUG test result, poor triceps skinfold thickness, normal grip strength, triglycerides 1.9 mmol/L, albumin 30 g/L), with a predicted malnutrition risk of 84.65%.
